# Self-reactivity of CD8 T-cell clones determines their differentiation status rather than their responsiveness in infections

**DOI:** 10.3389/fimmu.2022.1009198

**Published:** 2022-10-06

**Authors:** Darina Paprckova, Veronika Niederlova, Alena Moudra, Ales Drobek, Michaela Pribikova, Sarka Janusova, Kilian Schober, Ales Neuwirth, Juraj Michalik, Martina Huranova, Veronika Horkova, Michaela Cesnekova, Michaela Simova, Jan Prochazka, Jana Balounova, Dirk H. Busch, Radislav Sedlacek, Martin Schwarzer, Ondrej Stepanek

**Affiliations:** ^1^ Laboratory of Adaptive Immunity, Institute of Molecular Genetics of the Czech Academy of Sciences, Prague, Czechia; ^2^ Faculty of Science, Department of Cell Biology, Charles University, Prague, Czechia; ^3^ Laboratory of Immunity & Cell Communication, BIOCEV, First Faculty of Medicine, Charles University, Vestec, Czechia; ^4^ Institute for Medical Microbiology, Immunology, and Hygiene, Technical University of Munich, Munich, Germany; ^5^ Mikrobiologisches Institut – Klinische Mikrobiologie, Immunologie und Hygiene, Universitätsklinikum Erlangen, Friedrich-Alexander-Universität (FAU) Erlangen-Nürnberg, Erlangen, Germany; ^6^ Czech Centre for Phenogenomics, Institute of Molecular Genetics of the Czech Academy of Sciences, Vestec, Czechia; ^7^ Laboratory of Gnotobiology, Institute of Microbiology of the Czech Academy of Sciences, Novy Hradek, Czechia

**Keywords:** T cell, self-reactivity, antigen-inexperienced memory-like CD8 T cells, T-cell diversity, interferon response

## Abstract

Mature T cells are selected for recognizing self-antigens with low to intermediate affinity in the thymus. Recently, the relative differences in self-reactivity among individual T-cell clones were appreciated as important factors regulating their fate and immune response, but the role of self-reactivity in T-cell biology is incompletely understood. We addressed the role of self-reactivity in T-cell diversity by generating an atlas of mouse peripheral CD8^+^ T cells, which revealed two unconventional populations of antigen-inexperienced T cells. In the next step, we examined the steady-state phenotype of monoclonal T cells with various levels of self-reactivity. Highly self-reactive clones preferentially differentiate into antigen-inexperienced memory-like cells, but do not form a population expressing type I interferon-induced genes, showing that these two subsets have unrelated origins. The functional comparison of naïve monoclonal CD8^+^ T cells specific to the identical model antigen did not show any correlation between the level of self-reactivity and the magnitude of the immune response.

## Introduction

T cells recognize fragments of antigens presented by MHC molecules to trigger adaptive immune responses. Early studies have focused on how the T-cell antigen receptor (TCR) affinity for the cognate pMHC regulates the type and magnitude of the T-cell response (1, 2). However, individual T-cell clones differ not only in their antigenic specificity and affinity, but also in the strength of the interaction between their TCRs and self-pMHCs. The negative and positive selection of developing thymocytes sets lower and upper limits for the self-reactivity of mature T cells (3), which is different for CD4^+^ and CD8^+^ T-cell subsets (4).

It has been established that strongly self-reactive clones differentiate into regulatory T cells or unconventional T-cell subsets in the thymus [reviewed in (5)]. Recently, it has been documented that the relative level of self-reactivity contributes to steady-state T-cell heterogeneity as well as to inter-clonal differences within the T-cell immune response in the periphery [reviewed in (6)]. In helper CD4^+^ T cells, the relative level of self-reactivity determines the primary and memory T-cell responses (7) and their differentiation into Th1 *vs*. Tfh effector subsets (8).

Two major roles of the relative level of self-reactivity have been described in cytotoxic CD8^+^ T cells. First, it has been proposed that naïve CD8^+^ T cells with a relatively high self-reactivity undergo stronger foreign antigen-triggered expansion than their less self-reactive counterparts (9, 10). The first study compared the response of polyclonal CD8^+^ T cells expressing very high or very low levels of CD5, a proxy marker for self-reactivity in steady-state T cells, in *Listeria* infection (9). The potential limitation of this approach is the inability to control for prior quantitative (frequency) and qualitative (affinity) differences in pathogen-specific cells between the analyzed subsets. The second study compared three *Toxoplasma*-specific CD8^+^ T-cell clones to conclude that the clone with the lowest peripheral expression of two markers of self-reactivity, CD5 and Nur77-GFP reporter, showed the weakest antigenic response (10). The caveats of this study were the low number of analyzed clones and the fact that the weakly responsive clone had also the lowest affinity to the cognate *Toxoplasma* antigen.

The other described role of self-reactivity in the CD8^+^ T cell compartment is the spontaneous differentiation of highly self-reactive clones into antigen-inexperienced memory-like T cells (AIMT; alias virtual or innate memory T cells) (11–13). The AIMT cells show unique gene expression signatures and functions compared to canonical naïve T cells. AIMT cells rapidly produce IFNγ upon antigenic or IL-12/18 signaling (13, 14), show a higher level of tolerance in a type I diabetes model than naïve T cells (11), and have the ability to efficiently infiltrate prostate tumors (15). Additionally, highly self-reactive T cells were shown to give rise to a population of Ly6C^+^ naïve CD8^+^ T cells with upregulated type I interferon (IFN) response signature (16). As it has been previously shown that the type I IFN signaling pathway is important for AIMT cell differentiation (17), these cells are likely precursors of AIMT cells (16). Recently, a cluster of human T cells with upregulated type I IFN responsive genes has been described and named IFN signaling–associated gene high (ISAG^hi^) T cells (18). However, the relationship between the mouse Ly6C^+^ CD8^+^ T cells and human ISAG^hi^ T cells is unclear.

In this study, we used a collection of related mouse CD8^+^ T-cell clones with different levels of self-reactivity to assess the role of self-reactivity in T-cell fate commitment and immune response.

## Results

### Identification of two antigen-inexperienced T-cell subsets by single cell transcriptomics

**Figure 1:**
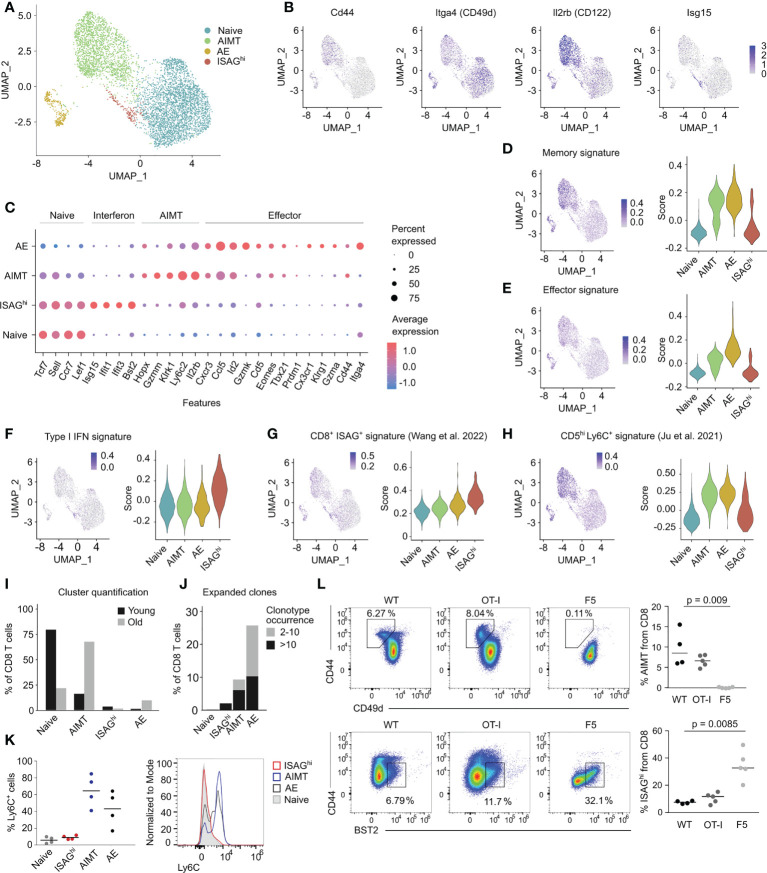
Transcriptomic analysis of murine CD8^+^ T cells. **(A)** UMAP projection of FACS-sorted CD8^+^ T cells from peripheral blood of three young and three aged C57BL/6J mice processed by scRNAseq. The cells from mice with the corresponding age were first pooled and labeled with barcoded antibodies. Subsequently, cells from young and aged mice were pooled prior to the scRNAseq. Cells are colored by manual annotations. **(B)** UMAP projections showing the expression of selected genes. **(C)** Expression of the signature genes of naïve, ISAG^hi^, AIMT and effector cells. Color of the dot indicates the average expression, size indicates the percentage of cells with non-zero expression. **(D–H)** Signature scores showing the expression of different gene modules in naïve, AIMT, AE, and ISAG^hi^ cells. **(D)** Expression of the module of immune memory genes expressed uniquely in CD8^+^ memory T lymphocytes (compared with effector or naïve cells), MSigDB # M10845 (19). **(E)** Expression of the module of genes down-regulated in naïve CD8^+^ T cells versus effector CD8^+^ T cells, MSigDB # M3036 (20). **(F)** Expression of genes from the GO pathway GO:0060338 “regulation of type I interferon-mediated signaling pathway”. **(G)** Expression of markers of human ISAG^+^ CD8^+^ T cells from the pan-cancer single cell T-cell atlas (21). **(H)** Expression of genes upregulated in murine CD5^hi^ Ly6C^+^ cells compared to CD5^low^ cells (16). **(I)** Comparison of the percentage of cells in each scRNAseq cluster in young versus aged mice. **(J)** Percentage of clonally expanded cells in each scRNAseq cluster. The count indicates the number of cells with the same TCRα CDR3 and TCRβ CDR3 nucleotide sequence. Cells with TCR receptors occurring only once in the dataset are not depicted. **(K, L)** Flow cytometry analysis of ISAG^hi^ and AIMT cells. **(K)** Quantification (left) and a representative histogram (right) of a flow cytometry analysis of Ly6C on naïve, ISAG^hi^, AIMT, and AE cells. n = 4 in two independent experiments. **(L)** Percentage of AIMT cells (top) and ISAG^hi^ cells (bottom) in WT mice and in transgenic mice with monoclonal OT-I and F5 TCRs. See the FMO control in **Figure S1M**. OT-I: n = 5, F5: n = 5, WT: n = 4. Three independent experiments. Medians are shown. P-value was calculated using the Kruskal-Wallis test.

To analyze the diversity of steady-state CD8^+^ T cells, we performed single-cell RNA sequencing (scRNAseq) on sorted blood CD8^+^ T cells from three young adult and three aged C57BL/6J mice housed in the specific pathogen-free (SPF) conditions. We identified six different cell clusters (**Figure S1A, Table S1**), which could be combined into four main subsets representing naïve cells, AIMT cells, antigen experienced (AE) cells, and a relatively small cluster of T cells expressing type I interferon-responsive genes (**Figure 1A**). The identity of the naïve, AIMT, and AE cells is documented by the abundance of transcripts encoding their established markers (naïve: CD44^low^, ITGA4^int^, IL2RB^low^; AIMT: CD44^hi^, ITGA4^-^, IL2RB^hi^; AE: CD44^hi^, ITGA4^hi^, IL2RB^int^) (**Figures 1B, C**) as well as by the overall expression of memory and effector signature genes (**Figures 1D, E**).

The cluster of interferon-responsive T cells was enriched for expression of genes involved in the regulation of type I interferon-mediated signaling pathway (**Figure 1F**, **Figure S1B**), such as *Isg15, Ifit1, Ifit2*, and *Bst2* (tetherin), but exhibited an overall naïve phenotype (**Figures 1B–E**). The gene expression of this cluster corresponded to the previously described human IFN signaling-associated gene high (ISAG^hi^) T cells (18, 21), but not to the mouse naïve Ly6C^+^ CD8^+^ T cells, which were also proposed to be interferon type I-induced (16) (**Figures 1G, H**). Because these cells are mouse counterparts of human ISAG^hi^ T cells, we refer to them as CD8^+^ ISAG^hi^ T cells henceforth.

To validate and extend the findings from scRNAseq, we used one of the markers of ISAG^hi^ cells, BST2 (**Figure S1C**), to identify ISAG^hi^ cells as CD44^low^ BST2^+^ by flow cytometry (**Figures S1D, E**). ISAG^hi^ cells were more frequent in the mesenteric lymph nodes (mLN) than in the spleen (**Figure S1F**) and their percentage was comparable to our scRNAseq data (**Figure 1I**). RT-qPCR analysis of sorted BST2^+^ cells confirmed increased expression of markers of ISAG^hi^ cells indicating that the sorted cells correspond to the scRNAseq profile of ISAG^hi^ cells (**Figures S1G–I**).

A combination of scRNAseq and flow cytometry allows us to characterize ISAG^hi^ cells in different contexts. First, unlike AIMT cells, which accumulate in aged mice and are enriched for expanded, probably self-reactive, clones (11, 14, 15, 22, 23), the frequency of naïve and ISAG^hi^ CD8^+^ T cells declines with age and their TCR repertoire is relatively diverse (**Figures 1I, J**, **Figures S1J–L**). Second, flow cytometry analysis confirmed the lack of cell surface expression of Ly6C in ISAG^hi^ and naïve CD8^+^ cells (**Figure 1K**), which distinguishes them from both AIMT cells and from recently reported naïve Ly6C^+^ CD8^+^ T cells, which were proposed to experience tonic type I IFN signaling (16). As we did not observe these naïve Ly6C^+^ CD8^+^ T cells as a separate cluster and because it was previously suggested that these cells are precursors of AIMT cells (16), it is very plausible that these cells are included in the AIMT cluster in our scRNAseq data (**Figure 1A**).

To further characterize AIMT and ISAG^hi^ T cells, we searched for them in two TCR transgenic mouse lines. AIMT cells are present in relatively highly self-reactive OT-I Rag2^-/-^ mice, but not in little self-reactive F5 Rag2^-/-^ mice (11) (**Figure 1L**). In contrast, ISAG^hi^ T cells were found at higher frequency in F5 Rag2^-/-^ mice than in OT-I Rag2^-/-^ mice (**Figure 1L**, **Figure S1M**). This indicated that ISAG^hi^ CD8^+^ T cells are not preferentially formed from highly self-reactive T-cell clones and might be even enriched in clones with relatively low level of self-reactivity.

Finally, ISAG^hi^ T cells are present in germ-free mice, excluding commensal bacteria as their major inducers (**Figure S1N**). Overall, it seems that these two non-canonical types of antigen-inexperienced cells, i.e., AIMT cells and CD8^+^ ISAG^hi^ T cells, are unrelated.

### A panel of related T-cell clones for studying the role of self-reactivity in T-cell biology

We have previously generated a collection of H2-K^b^-SIINFEKL (OVA)-reactive TCRs by sorting OVA tetramer-positive naïve and AIMT CD8^+^ T cells from Vβ5 transgenic mice with fixed TCRβ chain (11). These TCRs are very similar in that they bind K^b^-OVA, share the TCRβ chain, and use the TCR Vα2^+^ (TRAV14) chain. An important feature of these TCRs is that they were cloned from unchallenged pre-immune mice, which excludes any potential bias through enrichment of those clones which are good responders to the antigenic stimulation.

**Figure 2:**
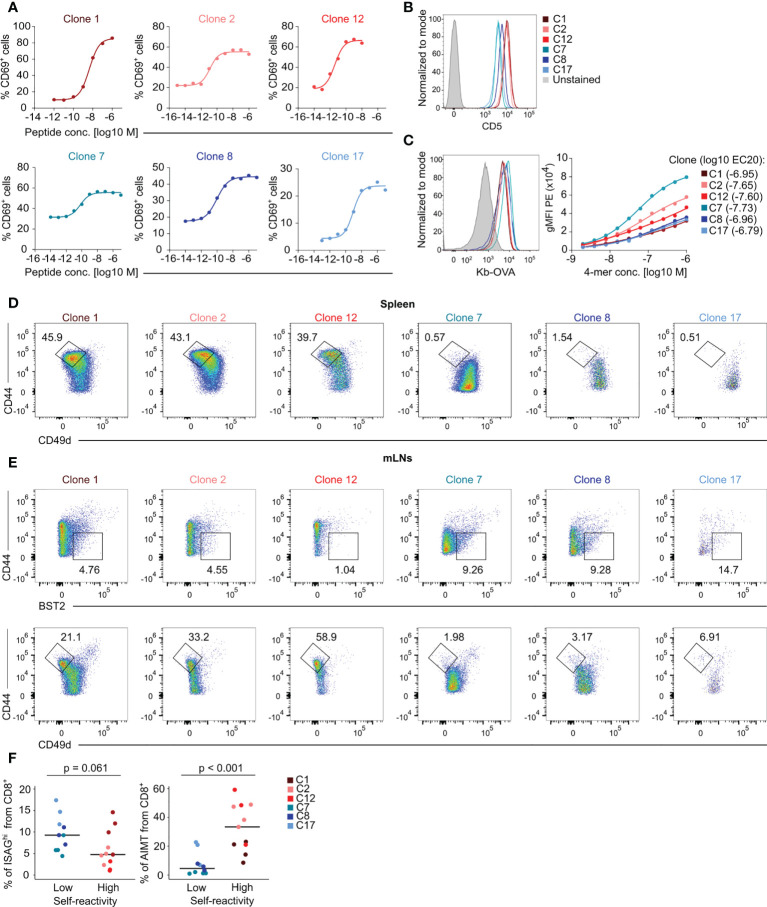
Characterization of the specificity, self-reactivity, and phenotype of a collection of monoclonal T cells. **(A)** Jurkat CD8^+^ OT-I TCRβ cells expressing indicated TCRα clones were activated by T2-Kb cells loaded with indicated concentrations of OVA peptide overnight. The level of CD69 was measured by flow cytometry. Curves were fit using nonlinear regression. **(B–E)** Peripheral lymphocytes were isolated from sub-lethally irradiated Ly5.1 mice transplanted with immortalized hematopoietic stem cells transduced with indicated clones and the recipient Ly5.2 CD8^+^ T cells were analyzed by flow cytometry. **(B)** Expression of the surface marker CD5 on LN T cells. A representative experiment out of three in total. **(C)** Left **–** staining of LN CD8^+^ T cells with fluorescently labeled K^b^-OVA tetramer. A representative experiment out of three in total. Right – staining with a dilution series of fluorescently labeled K^b^-OVA tetramer. Tetramer binding was measured by flow cytometry. Curves were fit using nonlinear regression. Averages from three independent experiments. **(D)** Representative FACS plots showing the percentage of AIMT (CD44^high^ CD49d^low^) T cells among CD8^+^ splenocytes from each clone. **(E)** Representative FACS plots of ISAG^hi^ T cells (CD44^low^ BST2^+^) and AIMT cells (CD44^high^ CD49d^low^) from each clone from mLNs. **(F)** Quantification of data from three independent experiments. Clone 1: n = 4, Clone 2: n = 4, Clone 12: n = 3, Clone 7: n = 4, Clone 8: n = 3, Clone 17: n = 3. Median is shown. The statistical analysis was performed using two-tailed Mann Whiney U test.

We selected six different TCR clones with confirmed H2-K^b^-OVA specificity when expressed in CD8^+^ Jurkat T cells (**Figure 2A**). We generated monoclonal T-cell populations expressing these TCRs using retrogenic mouse technology (**Figure S2A**) (11) to assess the role of self-reactivity in CD8^+^ T-cell biology. Based on the expression of CD5, we established the following hierarchy of self-reactivity among these clones: C1 ~ C2 ~ C12 > C8 > C7 ~ C17 (**Figure 2B**). Using the K^b^-OVA tetramer titration, we established the following hierarchy of affinity to K^b^-OVA among these clones: C7 > C2 > C12 > C1 ~ C8 ~ C17 (**Figure 2C**). Self-reactivity and affinity to the cognate antigen are independent features in our T-cell clone panel (**Figure S2B**). Although we observed variable surface TCR levels among the clones (**Figure S2C**), these inter-clonal differences did not strongly correlate with the affinity to K^b^-OVA (**Figure S2D**). In line with our previous data (11), only the three highly self-reactive clones formed a substantial number of AIMT cells (**Figure 2D**). In contrast, ISAG^hi^ T cells were formed more efficiently from clones with a low level of self-reactivity than from the highly self-reactive clones (**Figures 2E, F**), as observed with OT-I and F5 transgenic mice (**Figure 1L**).

### Self-reactivity does not predict the immune response of monoclonal T cells

**Figure 3:**
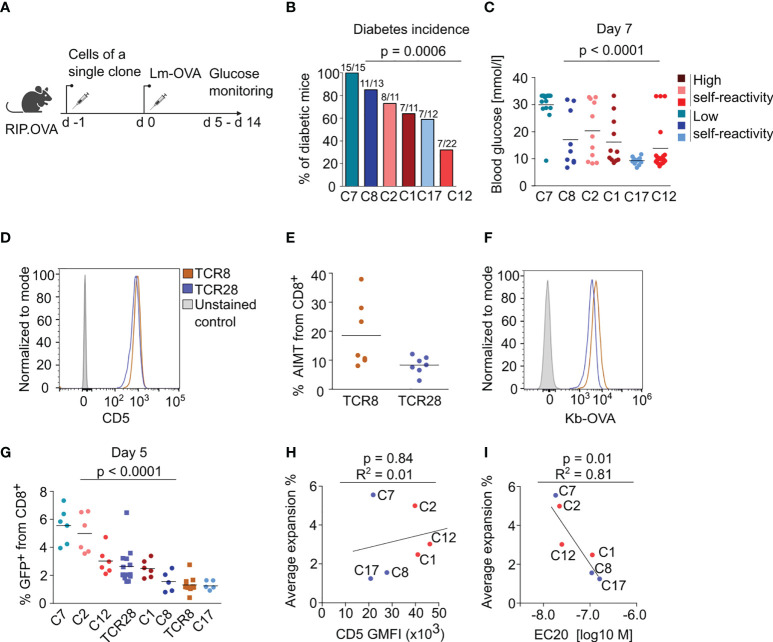
The self-reactivity does not dictate the magnitude of immune response of naïve CD8^+^ T cells. **(A-C)** 10^4^ CD8^+^ T cells from the indicated clone were transferred into RIP.OVA mice. The next day, mice were infected with Lm-OVA. Urine glucose levels were monitored on a daily basis on days 5-14 post infection. **(A)** Scheme of the experiment. **(B)** Percentage of the diabetic mice is shown. Number of the diabetic mice and total number of mice per group is indicated on top of each column. Three (C1, C2, C8, C17) or four (C7, C12) independent experiments. For the incidence of diabetes over time, see **Figure S3B**. **(C)** Glucose concentration in blood on day 7 post-infection. Mean. Clone 1: n=11, Clone 2: n= 11, Clone 7: n=15, Clone 8: n=13, Clone 12: n=22, Clone 17: n=12. **(D–F)** Phenotypic analysis of two additional monoclonal TCRs reactive to OVA, TCR8 and TCR28. Splenocytes and LN cells were isolated from sub-lethally irradiated Ly5.1 mice transplanted with the indicated clones and CD8^+^ T cells were analyzed by flow cytometry. **(D)** Expression of CD5 in CD8^+^ LN cells. A representative experiment out of three in total. **(E)** Percentage of AIMT (CD44^+^ CD49^-^) T cells among CD8^+^ splenocytes. Mean. n = 5 mice per group from two independent experiments. **(F)** Staining of monoclonal LN cells with fluorescently labeled K^b^-OVA tetramer. A representative experiment out of three in total. **(G)** Monoclonal naïve (CD44^-^) CD8^+^ T cells were sorted from LN cells and adoptively transferred to polyclonal Ly5.1 host mice (10 000 cells/mouse), which were infected one day later with transgenic Lm-OVA. Five days after the infection, the percentage of adoptively transferred GFP^+^ T cells among all CD8^+^ T cells was determined by flow cytometry. Mean. n = 6-12 per group from three independent experiments. The statistical significance was calculated using the Kruskall-Wallis test. **(H, I)** A linear fit between average expansion and **(H)** CD5 gMFI or **(I)** EC20 of the tetramer staining. The Pearson correlation coefficient and p value is shown.

We took advantage of our collection of K^b^-OVA-reactive clones to address the previously observed correlation between the clonal peripheral response and the level of self-reactivity in naïve T cells (9, 10). First, we assessed the ability of sorted naïve CD44^low^ T cells from the monoclonal populations to induce autoimmune diabetes in RIP.OVA mice upon priming with transgenic *Listeria monocytogenes* expressing OVA (Lm-OVA) (24) (**Figure 3A**, **Figure S3**). Although we observed differences between individual clones in this assay, there was no overall difference between the clones with high and low level of self-reactivity (**Figures 3B, C**, **Figure S3B**). Clone 7, which has the highest affinity to K^b^-OVA, was the most potent in inducing diabetes.

As the major readout in the previous studies investigating this question was the T-cell expansion, we assessed the expansion of our individual clones during Lm-OVA infection. We extended our T-cell clone collection with two more K^b^-OVA-specific clones, TCR8 and TCR28, which share the same TCRα chain and differ only in a single amino acid in the TCRβ CDR3 (**Figure S3C**) (25). TCR8 is more self-reactive than TCR28 as it expresses higher CD5 levels and forms more AIMT cells (**Figures 3D, E**). Moreover, TCR8 has also higher affinity to K^b^-OVA than TCR28 (**Figure 3F**) (25). To assess the proliferative response of these clones during cognate infection, we adoptively transferred naïve CD44^low^ monoclonal T cells into congenic Ly5.1 mice and infected them with Lm-OVA (**Figure S3A**). We quantified the expansion of monoclonal T cells on day 5 post-infection as the percentage of these cells out of all CD8^+^ T cells. We observed that individual T-cell clones show different level of expansion (**Figure 3G**). However, we observed no correlation between the expansion and the levels of self-reactivity (**Figure 3H**). Instead, we observed a correlation between the expansion and affinity to OVA in the original series of our six clones (**Figure 3I**). The clones TCR8 and TCR28 were not included in these comparisons (**Figures 3H, I**), because we measured their self-reactivity and K^b^-OVA binding only later, separately from the original six clones. The formation of KLRG1^+^ effector T cells was also variable among the clones, but it did not show any dependence on the level of self-reactivity (**Figure S3D**).

Overall, the analysis of the eight different K^b^-OVA-specific clones did not support the hypothesis that the level of self-reactivity predisposes T cells for expansion during cognate infection.

### The dependency of individual T-cell clones on the thymoproteasome

A possible explanation of the discrepancy between our data and previously published data concerning the role of self-reactivity in the immune response (9, 10) was that the previous reports studied T cells with extremely low or even zero level of self-reactivity in the periphery. One example of such cells can be T cells recognizing only such self-antigens that are processed by thymoproteasome in cortical thymic epithelial cells. To test this possibility, we generated a thymoproteasome-deficient *Psmb11^-/-^* mouse (**Figure S4A**). These mice exhibited a substantially reduced CD8^+^ T-cell compartment and enrichment for lymphopenia-induced AIMT cells (**Figures S4B, C**) as described previously (26, 27). We also observed increased numbers of NKT cells, effector NKT cells and γδ T-cells in *Psmb11^-/-^* (**Figures S4D, E**), probably as a consequence of high IL-7 and/or IL-15 availability caused by the reduction of CD8^+^ T cells.

**Figure 4:**
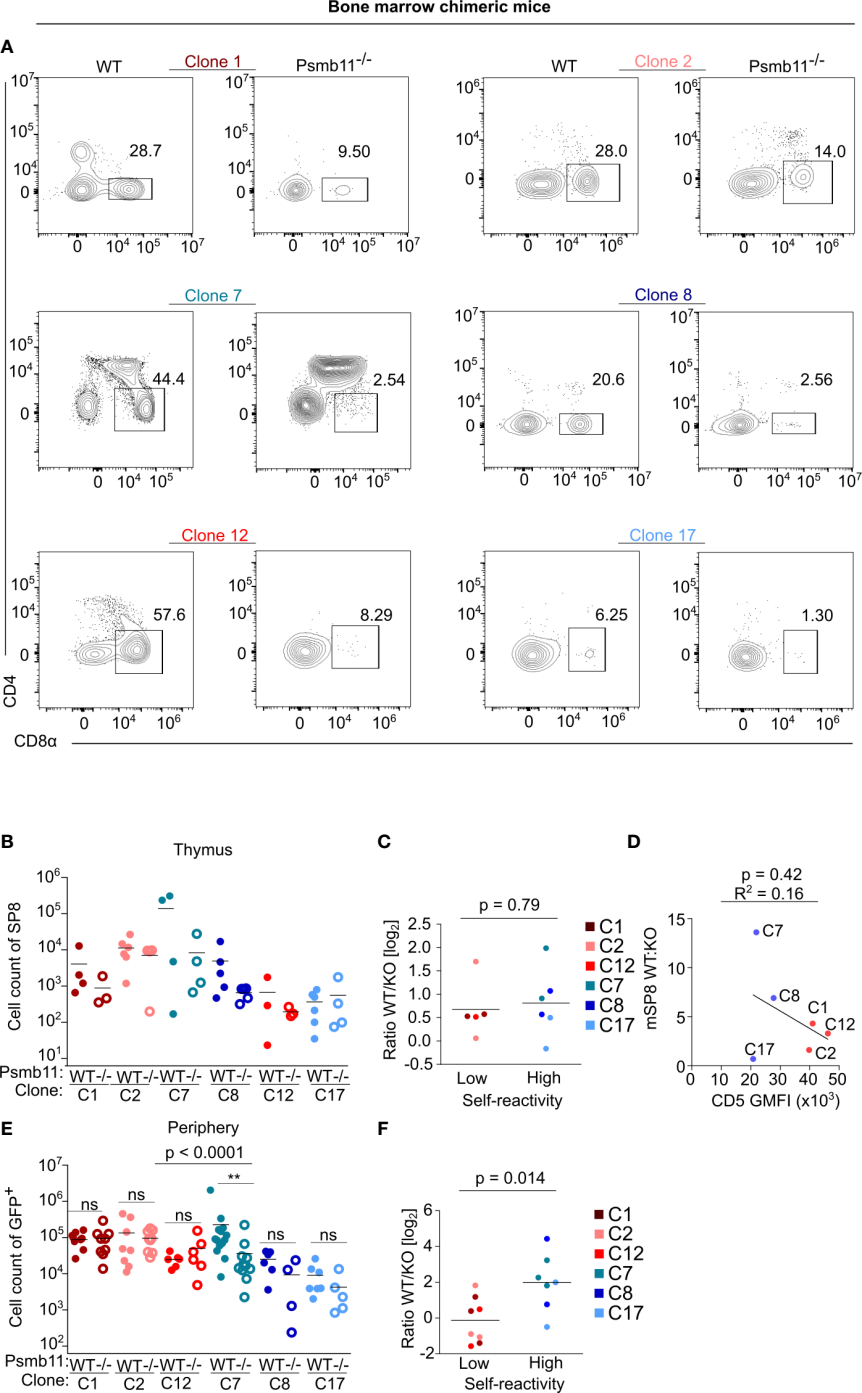
Generation of bone marrow chimeras of the clones in thymoproteasome deficient mice. WT or *Psmb11^-/-^* mice were sub-lethally irradiated and transplanted with hematopoietic stem cells transgenic for the indicated clones. Eight weeks later, thymi and LNs were analyzed by flow cytometry. n = 4-8 mice per group from three independent experiments. **(A)** Representative FACS plots showing thymic mature SP8 for all of the clones in WT or *Psmb11^-/-^* mice. **(B)** Numbers of donor mature SP8 in thymi of WT or *Psmb11^-/-^* mice. Mean. n = 4-5 mice per group from two independent experiments. **(C)** Numbers of donor CD8^+^ T cells isolated from thymi of WT or *Psmb11^-/-^* mice depicted as a ratio between WT and Psmb11^-/-^ (KO). Median. The statistical analysis was performed using the two-tailed Mann Whitney U test. **(D)** A linear fit between the WT : KO ratio of mature SP8 cells and CD5 gMFI levels. **(E)** Numbers of donor CD8^+^ T cells isolated from LNs of WT or *Psmb11^-/-^* mice. n=4-8 mice per group from three independent experiments. The statistical analysis was performed using Kruskal–Wallis test with Dunn’s post-tests. **(F)** Numbers of donor CD8^+^ T cells isolated from LNs of WT or *Psmb11^-/-^* mice depicted as a ratio between WT and Psmb11^-/-^ (KO). Median. The statistical analysis was performed using the two-tailed Mann Whitney U test. ** p<0.01, ns (not significant) p>0.05.

We compared WT (*Psmb11^+/+^* or *Psmb11^+/-^*) and *Psmb11^-/-^* as donors for the transplantation of hematopoietic stem cells transduced with retroviral vectors encoding the TCR clones. There was a variable dependence of the positive selection on the thymoproteasome among the clones in these bone marrow chimeras (**Figures 4A, B**). Unlike the previous studies using a few unrelated clones (27, 28), we did not see a strong correlation between the self-reactivity and thymoproteasome dependence (**Figure 4C**). The situation changed in the periphery. The absolute numbers of highly self-reactive clones in the LNs were comparable in WT and *Psmb11* hosts, whereas the numbers of weakly self-reactive clones were higher in WT than *Psmb11^-/-^* recipients (**Figures 4D–F**). This can be explained by the lymphopenic environment in *Psmb11^-/-^* hosts (**Figure S4G**), which preferentially stimulates the homeostatic proliferation of highly self-reactive clones (6). Indeed, the analysis of the percentage of the donor cells among all LN CD8^+^ T cells suggested the peripheral homeostatic expansion of the highly, but not the lowly, self-reactive clones (**Figure S4H**). Overall, although most of the tested clones showed some dependence on the thymoproteasome, none of them was completely thymoproteasome-dependent as shown for some other clones such as HY TCR transgenic T cells (27, 28). For this reason, we cannot exclude the possibility that clones which are absolutely dependent on thymoproteasome-processed peptides, and thus have zero self-reactivity in the periphery, have substantially impaired proliferative responses during infection.

Overall, our data indicate that there is no general relationship between the self-reactivity of naïve CD8^+^ T cell clones and their proliferative response during infection.

## Discussion

Recently, the relative level of self-reactivity has been identified as an important factor regulating peripheral T cells (6). It regulates the proliferation and differentiation of naive T cells during the immune response (9, 10, 29) as well as spontaneous differentiation of naive CD8^+^ T cells into AIMT cells (11, 12) and Ly6C^+^ cells with reported tonic type I interferon signaling (16).

In this study, we generated an atlas of mouse blood CD8^+^ T cells to resolve the unclear relationship between unconventional antigen-inexperienced subsets: CD8^+^ AIMT cells (13, 30) and two CD8^+^ T-cell subsets with upregulated type I interferon responsive genes (16, 18). By comparing our and previously published data (16), we found out that naive CD8^+^ Ly6C^+^ T cells have a gene expression profile largely corresponding to AIMT cells, which is consistent with the idea that they represent AIMT cell precursors (16). On the contrary, previously described ISAG^hi^ T cells represent a subset of naive CD8^+^ T cells, which is unrelated to AIMT and Ly6C^+^ T cells. The individual T-cell clones with high or low self-reactivity preferentially differentiate into AIMT or ISAG^hi^ T cells, respectively. The origin of ISAG^hi^ T cells is unclear. Given their expression of type I IFN-induced genes and their relative abundance in mLNs, it is plausible that they are induced by the type I IFN produced in the gut. However, this idea is challenged by normal frequencies of ISAG^hi^ CD8^+^ T cells in germ-free mice, which were previously shown to produce less type I IFN than SPF mice (31). It is thus plausible that ISAG^hi^ T cells are induced locally by a constitutive tissue- or cell-type specific production of type I IFNs, which is independent of microbial colonization, and/or these cells arise in a type I IFN-independent manner.

To address the role of self-reactivity in T-cell biology, we used a collection of six related OVA-specific T-cell clones with variable levels of self-reactivity. The major advantage of this collection was that these clones were isolated from pre-immune repertoire and that they were comparable to each other in many aspects, such as TCRβ chain, TRAV gene usage, and antigen specificity. We confirmed our previous findings that relatively highly self-reactive CD8^+^ T-cell clones generated AIMT cells (11). In contrast, these highly self-reactive clones were slightly less efficient than weakly self-reactive clones in the formation of ISAG^hi^ T cells. The probable explanation is that their commitment to AIMT cells is mutually exclusive with the differentiation into ISAG^hi^ T cells.

In the next step, we used our TCR panels extended by two OVA-specific clones (25) to test whether highly self-reactive clones are predisposed to a stronger immune response as proposed previously (9, 10). In contrast to these reports, we did not observe a significant correlation between the level of self-reactivity and the expansion during *Listeria* infection or in an OVA-dependent diabetic model as the variability of the clones was primarily driven by the differences in their apparent affinity to their cognate antigen. In the infection model, we analyzed the progeny of the adoptively transferred monoclonal T cells only on day five post-infection. We have chosen this time-point because there is already a detectable expansion, but it is still before it peaks. Thus, these time points should not be much influenced by extrinsic factors potentially limiting the maximal expansion. Although we cannot formally exclude that some self-reactivity-driven intra-clonal differences could be observed only at later time-points, we find this relatively unlikely as the hypothetical differences among T-cell clones caused by a differential level of tonic signaling in the steady-state would manifest rather earlier than later during the course of the cognate infection.

There are multiple possible explanations why our results did not support the previous studies. The study by Swee et al. investigated only three different clones, and their conclusions are largely based on one poorly responding clone, which also had the lowest affinity to the cognate antigen (10). The study by Fulton et al. focused on responses of polyclonal T cells (9). However, it is not possible to control for the prior frequency of antigen-specific T cells in the weakly and strongly self-reactive groups. It has been proposed that highly self-reactive clones are more likely to recognize a foreign cognate antigen than weakly self-reactive clones (32). For this reason, or just based on a stochastic basis, the group of highly self-reactive clones might be enriched for clones specific to the model pathogen.

However, there is an additional possible explanation of these discrepancies. It has been shown that the positive selection of some very weakly self-reactive CD8^+^ T-cell clones is dependent on self-peptides generated by thymoproteasome (27, 28). These T-cell clones have effectively null self-reactivity in the periphery and might show impaired immune responses. Indeed, the level of self-reactivity of the poorly responding clone in the study by Swee et al. dropped when the cells matured and migrated from the thymus to the periphery (10). For this reason, we generated thymoproteasome-deficient *Psmb11^-/-^* mice to test the role of thymoproteasome in the thymic maturation of our set of clones. We observed that our T-cell clones have different levels of thymoproteasome dependence, but none of the clones showed a complete developmental block in the absence of the thymoproteasome. Interestingly, clone C7, which was the most potent in the *in vivo* immune assays, was affected by the thymoproteasome deficiency the most. Overall, we did not find evidence supporting the possible explanation of the previous observations by impaired immune response of strictly thymoproteasome-dependent T-cell clones. However, we cannot formally exclude it, since all our clones showed only partially blocked thymic development in the *Psmb11^-/-^* mice.

Altogether, our data show that the level of self-reactivity of CD8^+^ T cells regulates their differentiation into AIMT cells (exclusively from highly self-reactive clones) and ISAG^hi^ T cells (preferentially from intermediate and weakly self-reactive clones). It has been previously shown that these two unconventional subsets of antigen-inexperienced T cells have different functional properties compared to classical naïve T cells (11–13, 15, 18). In contrast, the analysis of our set of OVA-reactive clones does not suggest that the level of self-reactivity strongly determines how naïve T cells respond during the immune response.

## Materials and methods

### Mice

All mice had C57BL/6J background. If not indicated, 6-12 weeks old mice were used. Males and females were used. Age- and sex-matched pairs of animals were used in the experimental groups. If possible, littermates were equally divided into the experimental groups. Mice were bred in the SPF facility (Institute of Molecular Genetics of the Czech Academy of Sciences) or in germ-free and conventional facilities (Laboratory of Gnotobiology, Institute of Microbiology of the Czech Academy of Sciences) in accordance with the laws of the Czech Republic. Germ-free animals were kept in Trexler-type plastic isolators, food (V1126-000, Ssniff, Soest, Germany) and poplar wood granulate bedding (Safe select fine, Safe, Germany) were irradiated (50 kGy, Bioster) and water was autoclaved. Axenicity of animals was verified regularly as previously described (33). Animal protocols were approved by the Resort Professional Commission for Approval of Projects of Experiments on Animals of the Czech Academy of Sciences, Czech Republic. Ly5.2, Ly5.1 (34), RIP.OVA (35), OT-I Rag2^-/-^ (36, 37), and Vβ5 (38) strains were described previously. Mice were kept in the animal facility with 12 hours of light and dark cycle with food and water ad libitum.

*Psmb11^-/-^* mice were generated on C57BL/6N background in the Czech Centre for Phenogenomics, Institute of Molecular Genetics of the Czech Academy of Sciences. The mice were generated by pronuclear microinjection of Cas9 mRNA and sgRNA into one-cell-stage murine embryos as described previously (39). sgRNA sequences with the PAM motif in bold (3′ end) were as follows:

sgRNA target 1: GATGTCCTGCTACCGCGGTCTGG
sgRNA target 2: TCGTCACGGTGTCATCGCTGCGG

The founders were back-crossed on C57BL/6J background for at least five generations. Genotyping primers used for detection of the mutations were as follows: FW primer 5’-AGGTGGCGTCTTCAGAGTGT paired with RV primer 5’-AGGGTGTAGGCTTCCTGGAT.

### Antibodies and reagents

Antibodies to following antigens were used for flow cytometry: anti-CD4 (clone RM4-5, Biolegend #100536), anti-CD5 (clone 53-7-3, Biolegend #100627, #100625), anti-CD8α (clone 53-6.7, Biolegend #100738), anti-CD24 (clone M1/69, Biolegend #101806), anti-CD25 (clone PC61, Biolegend #102016 and #102036), anti-CD44 (clone IM7, Biolegend #103049), anti-CD45.1 (clone A20, Biolegend #110723), anti-CD45.2 (clone 104, Biolegend #109808), anti-CD49d (clone R1-2, Biolegend #103618), anti-CD127 (clone A7R34, Biolegend #135012), anti-TCRβ (clone H57-597, Biolegend #109218), anti-KLRG1 (clone 2F1, Biolegend #138421), anti-Ly6C (clone HK1.4, Biolegend #128006), biotin-conjugated anti-BST2 (clone 927, Biolegend #127006), anti-biotin (clone 1D4-C5, Biolegend #409004). Viability was stained with LIVE/DEAD™ Fixable Near-IR Dead Cell Stain Kit (Invitrogen #L34975), Zombie Green™ Fixable Viability Kit (Biolegend, #423111) or Hoechst 33258 (Invitrogen, #H3569). For the analysis of Jurkat cell lines, anti-CD8 (clone MEM-31, Exbio #1P-207-T025), anti-CD69 (clone FN50, Exbio #T7-552-T100) were used. Antibodies were conjugated with various fluorophores and used according to the manufacturer’s instructions.

### Cell counting and cell culture

Cells were counted using Z2 Coulter Counter Analyzer (Beckman Coulter) or Cytek Aurora flow cytometer (Cytek).

Jurkat cells (parental line RRID : CVCL_0367) were cultured at 37°C/5% CO_2_ in RPMI (Sigma Aldrich), supplemented with 10% FBS (GIBCO), 100 U/mL penicillin (BB Pharma), 100 mg/mL streptomycin (Sigma Aldrich), 40 mg/mL gentamicin (Sandoz).

### Generation and analysis of mouse TCR expressing Jurkat T cells

1×10^6^ of Jurkat T cells with TCR OTI-β and human CD8αβ (40) were transduced with TCRα genes in MSCV-ires-GFP vectors as described previously (40). Briefly, Jurkat cells were resuspended in MSCV pseudovirus-containing supernatant supplemented with 8 μg/mL of polybrene and centrifuged (45 min, 1200 g, 30°C). Cells were incubated at 37°C overnight. On day 2, cells were transferred into a 15 ml falcon tube, centrifuged (10 min, 400 g), and resuspended in fresh medium. On day 4, cells were transferred from six-well plate to the tissue culture flasks to expand further. Transduced cells were sorted as GFP^+^ positive on the BD Influx Cell Sorter (BD Biosciences) in the IMG core facility. T2-Kb cells were resuspended in RPMI medium (Sigma Aldrich), stained with DDAO dye (ThermoFisher Scientific) and incubated at 37°C for 10 min. Cells were washed in RPMI medium and centrifuged (5 min, 500 g). 2 × 10^5^ of T2Kb cells were re-suspended in 100 μL RPMI media with indicated concentrations of OVA peptide (SIINFEKL, storage concentration 2mM, Eurogentec) and incubated at 37°C for 2 hours.

After 2 hours, T2-Kb cells were mixed with 1 × 10^5^ transgenic Jurkat cells and incubated overnight (total volume 200 μL). The next day, the cells were stained with α-huCD69-PE-Cy7 (Biolegend) on ice for 45 minutes and analyzed by flow cytometry.

### Flow cytometry and cell sorting

For the surface staining, cells were incubated with diluted antibodies in FACS buffer (PBS, 2% FBS, with or without 1 mM EDTA, 0.1% NaN_3_) on ice. LIVE/DEAD near-IR dye (Life Technologies) was used for discrimination of live and dead cells. For some experiments, enrichment of CD8^+^ T cells was performed using magnetic bead separation kits EasySep (STEMCELL Technologies) or Dynabeads (Thermo Fisher Scientific) *via* negative selection according to manufacturer’s instructions prior to the analysis or sorting by flow cytometry. Flow cytometry was carried out with Cytek Aurora flow cytometer (Cytek) or LSRII (BD Biosciences). Cell sorting was performed using FACSAria III or Influx (BD Bioscience). Data were analyzed using FlowJo software (TreeStar).

### Monoclonal retrogenic T cells

Generation of immortalized bone marrow hematopoietic stem cells was described previously (41). We have used Vβ5 Rag2^-/-^ NupHox cell line described previously (11). Two days after the transduction, GFP^+^ cells were FACS-sorted and transplanted into irradiated (6 Gy) congenic Ly5.1 recipient mice using X-RAD 225XL Biological irradiator (Precision X-Ray). At least 8 weeks after the transplantation, the recipient mice were sacrificed and donor thymic, LN, and splenic T cells were used for downstream analyses.

### Tetramer binding

K^b^-OVA tetramers were generated by incubating biotinylated pMHCI monomers (42) with PE-streptavidin (Invitrogen) at a 4:1 ratio on ice. Streptavidin was added in three doses with 20 min incubation on ice in between. For the tetramer titration experiments, peripheral T cells were isolated, incubated with serially diluted tetramers (1×10^-6^ to 2×10^-9^ M) for 20 min on ice. The supernatant was replaced with PBS + 2% FBS and cells were immediately analyzed using a sample cooling system. EC20 (concentration of tetramer resulting in 20% of the maximal achieved signal intensity) was calculated using non-linear regression in GraphPad Prism 5.0.

### Listeria infection

LN T cells were isolated from monoclonal retrogenic mice. FACS-sorted 1×10^4^ naïve (CD44^low^) CD8^+^ T cells were adoptively transferred to Ly5.1 congenic host mice. The following day, mice were injected with 5,000 CFU of transgenic Lm expressing OVA antigen. The expansion of the responsive cells was analyzed by flow cytometry on day 5 after the infection.

### Model of autoimmune diabetes

The model of experimental autoimmune diabetes has been described previously (11). Briefly, FACS-sorted 1×10^4^ naïve (CD44^low^) monoclonal retrogenic CD8^+^ T cells were adoptively transferred into a recipient RIP.OVA mouse i.v. On the following day, the recipient mice were immunized with Lm-OVA. Urine glucose was monitored on a daily basis using test strips (GLUKOPHAN, Erba Lachema). Blood glucose was measured using Contour blood glucose meter (Bayer) on a day 7 post-immunization. The animal was considered diabetic when the concentration of glucose in the urine reached ≥ 1,000 mg/dl for two consecutive days.

### RT-qPCR

Total RNA of 2-8×10^5^ FACS-sorted BST2^+^ or BST2^-^ cells was isolated using the RNEasy plus Micro kit (Quiagen, #74034) and transcribed into cDNA using RevertAid reverse transcriptase (Thermofisher, # EP0442) with oligo(dT)18 primers according to the manufacturer’s instructions. cDNA was used for quantitative PCR using the following primers: Isg15_FTCTGACTGTGAGAGCAAGCAG, Isg15_RCCTTTAGGTCCCAGGCCATTG, Ifit1_FAGGCAGGTTTCTGAGGAGTTC, Ifit1_RATCAGCATTCTCTCCCATGGTT, Bst2_FGTTGGCAGGTCACAGTTGTTT, Bst2_RGTTTCCACATCCTCAGGGCT, GAPDH_Ftgcaccaccaactgcttagc, GAPDH_Rggcatggactgtggtcatgag, Eef1a1_Facacgtagattccggcaagt, Eef1a1_Raggagccctttcccatctc, Tubb2A_Faaccagatcggcgctaagt, Tubb2A_Rtgccagcagcttcattgta. Samples for qPCR were measured by LightCycler 480 (Roche) in technical triplicates and the median value was used for further calculations. The expression of the indicated genes was normalized to the geometric mean of three reference genes (*Gapdh, Eef1a1, Tubb2a*) in the same sample.

### Single-cell RNA sequencing

Young (8 weeks old, n = 3) and aged (88 weeks old, n = 3) WT mice were anesthetized using an i.p. injection of Ketamine (100 mg/1 kg, Bioveta) Xylazine (10 mg/1kg, Rometar). Carotid artery blood was collected to individual EDTA-coated tubes (41.1504.015, Sarstedt.1504.015, Sarstedt) supplemented with 100 μL of 3.5 mM EDTA (#607-429-00-8, Penta). Blood from the three corresponding mice was pooled, centrifuged (4 °C, 400 g, 5 min) and the supernatant was removed. Cells were lysed in 5 mL of ACK buffer for 3 minutes in room temperature. The reaction was stopped by adding 10 mL PBS and the lysis step was repeated once more. Cells were stained on ice in darkness in 200 µL of PBS supplemented with 2% FBS and 1:400 diluted feature barcoding antibodies CD45-FB1 and CD45-FB2 (Biolegend #103102 conjugated to DNA oligos [AmC12]CGGAGATGTGT ATAAGAGACAGNNNNNNNNNNTGGTGACAAGTATCTNNNNNNNNNCCCATATAAGAAA, respectively [AmC12]CGGAGATGTGTATAAGAGACAGNNNN NNNNNNGGACGCAACTTAAGANNNNNNNNNCCCATATAAGAAA using Thunder-Link PLUS Oligo Conjugation System, #425-0300, Expedeon, according to the manufacturer’s instructions). After 10 min of staining, 1:200 diluted mouse anti-CD5 (#100625, BioLegend) and anti-CD8α (#100723, BioLegend) were added for additional 20 min. CD8α^+^ CD5^+^ cells were sorted using an Influx (BD Bioscience). The viability and concentration of cells after sort were measured using the TC20 Automated Cell Counter (#1450102, Bio-Rad). The viability of the cells pre-loading was higher than 90%.

For scRNAseq analysis, the samples were pooled together with cells coming from BALB/c mice that were analyzed for an unrelated experiment. These unrelated cells were labeled with different feature barcoding antibodies and removed during the analysis. Cells were loaded onto a 10x Chromium machine (10x Genomics) aiming at the yield of 1,000 cells per sample. The subsequent steps were done following the Feature Barcode technology for Cell Surface Protein protocol (#CG000186 Rev D) with the Chromium Single Cell 5’ Library & Gel Bead and Chromium Single Cell 5’ Feature Barcode Library kits (10x Genomics, #PN-1000014, #PN-1000020, #PN-1000080, #PN-1000009, #PN-1000084, #PN-1000071). Resulting cDNA libraries were sequenced on a NextSeq 500/550 (Illumina) with the High Output Kit v2 (150 cycles, Illumina, # FC-404-1002, Illumina).

### Analysis of scRNAseq data

The raw scRNAseq data were mapped to Mouse Reference GRCm38 obtained from Ensembl database v102 (43) by 10x Genomics Cell Ranger 5.0.0 with the default parameters (44). The V(D)J sequences were mapped by 10x Genomics Cell Ranger 5.0.0 to IMGT reference (45) pre-built in accordance with 10X Genomics instructions. The MiXCR software (46) was used to extract additional V(D)J sequences from gene expression data (“analyze shotgun” pipeline with default parameters). Initially, cells with less than 200 transcripts and/or more than 15% of transcripts mapping to mitochondrial genes and cells identified as doublets by V(D)J sequences (having more than 2 TCRα or 1 productive TCRβ/2 non-productive TCRβ sequences) were removed. Mitochondrial genes, TCRα and TCRβ V(D)J-genes, ribosomal genes and genes whose transcripts were detected in less than three cells were excluded. Log normalization (scale factor = 1 × 10^4^), scaling, identification of variable features (500 variable features), integration of the two datasets using canonical correlation analysis, dimensional reduction (PCA and UMAP using the top 24 principal components), identification of nearest neighbors and Louvain clustering (resolution = 0.5) were performed using the Seurat R package v4.0.3 (47) on R v4.0.4. Quality control (QC) included removal of low-quality clusters recognized by overall low counts of reads and detected genes with high percentage of mitochondrial genes and clusters containing contaminating cells. In total, 5,461 cells passed the QC steps.

The signature scores were calculated for each cluster using the AddModuleScore function from the Seurat package. Signature genes were selected from the Molecular Signatures Database v7.5.1 (Systematic names: #M10845 (19) for the memory signature, #M3036 (20) for the effector signature), from the Gene ontology pathways database (48) (GO:0060338 for the type I IFN signature), or from the literature (CD5hiLy6C+ signature (16), and CD8^+^ ISAG^+^ signature (21).

The code for the cell filtration to hashtags, barcode extraction and whole downstream analysis including V(D)J is accessible on GitHub (https://github.com/Lab-of-Adaptive-Immunity/mouse-cd8-scRNAseq).

## Data availability statement

The datasets presented in this study can be found in online repositories. The names of the repository/repositories and accession number(s) can be found below: https://www.ncbi.nlm.nih.gov/geo/, GSE208795.

## Ethics statement

The animal study was reviewed and approved by Resort Professional Commission for Approval of Projects of Experiments on Animals of the Czech Academy of Sciences, Czech Republic.

## Author contributions

DP, AM, AD, and SJ planned, performed, and analyzed *in vivo* experiments with monoclonal T cells. VN, AN, and JM generated CD8^+^ T cell scRNAseq atlas. VN compared our transcriptomic data with published datasets. VN and MC planned, performed, and analyzed experiments with ISAG^hi^ T cells. MP and MH cloned OVA-specific TCRs. MP and VH generated and characterized Jurkat cell lines expressing these model TCRs. MSc housed and provided germ-free mice. KS and DB characterized and provided TCR8 and TCR28 clones. JB, MSi, and RS generated *Psmb11^-/-^* mice. DP, JB, MSi, and RS phenotyped *Psmb11^-/-^* mice. OS conceived the study, analyzed data, and supervised the project. DP, VN, and OS wrote the draft of the manuscript. All authors contributed to the article and approved the submitted version.

## Funding

The project was supported by the Czech Science Foundation (19-03435Y to OS and 21-19640M to MS), the Czech Academy of Sciences (RVO 68378050 to OS), the National Institute of virology and bacteriology (Programme EXCELES, ID Project No. LX22NPO5103) - Funded by the European Union - Next Generation EU, Charles University Grant Agency (838120 to VN), and the Czech Ministry of Education, Youth and Sports and the European Regional Development Fund (LM2015040, LM2018126, OP RDI CZ.1.05/2.1.00/19.0395, OP RDI BIOCEV CZ.1.05/1.1.00/02.0109 to RS). The contribution by DB was supported by the Deutsche Forschungsgemeinschaft (DFG, German Research Foundation) SFB 1054/3 - 210592381 (project B09) and SFB- TRR 338/1 2021 - 452881907 (project A01).

## Acknowledgments

We thank Ladislav Cupak for technical assistance and mouse genotyping. We thank members of the IMG flow cytometry and animal facilities at the IMG and the Institute of Microbiology for their assistance. We thank teams of the Czech Centre for Phenogenomics at the IMG for generation of the mouse model and its phenotyping. DP and SJ are students of the Faculty of Science, Charles University in Prague.

## Conflict of interest

The authors declare that the research was conducted in the absence of any commercial or financial relationships that could be construed as a potential conflict of interest.

## Publisher’s note

All claims expressed in this article are solely those of the authors and do not necessarily represent those of their affiliated organizations, or those of the publisher, the editors and the reviewers. Any product that may be evaluated in this article, or claim that may be made by its manufacturer, is not guaranteed or endorsed by the publisher.

## References

[1] Zehn D Lee SY Bevan MJ. Complete but curtailed T cell response to very low affinity antigen. Nature (2009) 458(7235):211–4. doi: 10.1038/nature07657 PMC2735344 19182777

[2] King CG Koehli S Hausmann B Schmaler M Zehn D Palmer E. T Cell affinity regulates asymmetric division, effector cell differentiation, and tissue pathology. Immunity (2012) 37(4):709–20. doi: 10.1016/j.immuni.2012.06.021 PMC3622938 23084359

[3] Daniels MA Teixeiro E Gill J Hausmann B Roubaty D Holmberg K. Thymic selection threshold defined by compartmentalization of Ras/Mapk signalling. Nature (2006) 444(7120):724–9. doi: 10.1038/nature05269 17086201

[4] Horkova V Drobek A Mueller D Gubser C Niederlova V Wyss L. Dynamics of the coreceptor-lck interactions during T cell development shape the self-reactivity of peripheral Cd4 and Cd8 T cells. Cell Rep (2020) 30(5):1504–14. doi: 10.1016/j.celrep.2020.01.008 PMC7003063 32023465

[5] Stritesky GL Jameson SC Hogquist KA. Selection of self-reactive T cells in the thymus. Annu Rev Immunol (2012) 30:95–114. doi: 10.1146/annurev-immunol-020711-075035 22149933 PMC3518413

[6] Paprckova D Stepanek O. Narcissistic T cells: Reactivity to self makes a difference. FEBS J (2021) 288(6):1778–88. doi: 10.1111/febs.15498 32738029

[7] Persaud SP Parker CR Lo WL Weber KS Allen PM. Intrinsic Cd4(+) T cell sensitivity and response to a pathogen are set and sustained by avidity for thymic and peripheral complexes of self peptide and mhc. Nat Immunol (2014) 15(3):266–74. doi: 10.1038/ni.2822 PMC3944141 24487322

[8] Weber KS Li QJ Persaud SP Campbell JD Davis MM Allen PM. Distinct Cd4(+) helper T cells involved in primary and secondary responses to infection. P Natl Acad Sci USA (2012) 109(24):9511–6. doi: 10.1073/pnas.1202408109 PMC3386110 22645349

[9] Fulton RB Hamilton SE Xing Y Best JA Goldrath AW Hogquist KA. The tcr's sensitivity to self peptide-mhc dictates the ability of naive Cd8(+) T cells to respond to foreign antigens. Nat Immunol (2015) 16(1):107–17. doi: 10.1038/ni.3043 PMC4270846 25419629

[10] Swee LK Tan ZW Sanecka A Yoshida N Patel H Grotenbreg G. Peripheral self-reactivity regulates antigen-specific Cd8 T-cell responses and cell division under physiological conditions. Open Biol (2016) 6(11):160293. doi: 10.1098/rsob.160293 27881740 PMC5133449

[11] Drobek A Moudra A Mueller D Huranova M Horkova V Pribikova M. Strong homeostatic tcr signals induce formation of self-tolerant virtual memory Cd8 T cells. EMBO J (2018) 37:e98518. doi: 10.15252/embj.201798518 29752423 PMC6043851

[12] White JT Cross EW Burchill MA Danhorn T McCarter MD Rosen HR. Virtual memory T cells develop and mediate bystander protective immunity in an il-15-Dependent manner. Nat Commun (2016) 7:1–13. doi: 10.1038/ncomms11291 PMC4844673 27097762

[13] Haluszczak C Akue AD Hamilton SE Johnson LD Pujanauski L Teodorovic L. The antigen-specific Cd8+ T cell repertoire in unimmunized mice includes memory phenotype cells bearing markers of homeostatic expansion. J Exp Med (2009) 206(2):435–48. doi: 10.1084/jem.20081829 PMC2646575 19188498

[14] Moudra A Niederlova V Novotny J Schmiedova L Kubovciak J Matejkova T. Phenotypic and clonal stability of antigen-inexperienced memory-like T cells across the genetic background, hygienic status, and aging. J Immunol (2021) 206(9):2109–21. doi: 10.4049/jimmunol.2001028 PMC7610663 33858960

[15] Miller CH Klawon DEJ Zeng S Lee V Socci ND Savage PA. Eomes identifies thymic precursors of self-specific memory-phenotype Cd8(+) T cells. Nat Immunol (2020) 21(5):567–77. doi: 10.1038/s41590-020-0653-1 PMC7193531 32284593

[16] Ju YJ Lee SW Kye YC Lee GW Kim HO Yun CH. Self-reactivity controls functional diversity of naive Cd8(+) T cells by Co-opting tonic type I interferon. Nat Commun (2021) 12(1):6059. doi: 10.1038/s41467-021-26351-3 34663827 PMC8523551

[17] Martinet V Tonon S Torres D Azouz A Nguyen M Kohler A. Type I interferons regulate eomesodermin expression and the development of unconventional memory Cd8(+) T cells. Nat Commun (2015) 6:7089. doi: 10.1038/ncomms8089 25953241 PMC4432629

[18] Wang XF Shen XR Chen S Liu HY Hong N Zhong HB. Reinvestigation of classic T cell subsets and identification of novel cell subpopulations by single-cell rna sequencing. J Immunol (2022) 208(2):396–406. doi: 10.4049/jimmunol.2100581 34911770

[19] Goldrath AW Luckey CJ Park R Benoist C Mathis D. The molecular program induced in T cells undergoing homeostatic proliferation. Proc Natl Acad Sci U.S.A. (2004) 101(48):16885–90. doi: 10.1073/pnas.0407417101 PMC534746 15548615

[20] Luckey CJ Bhattacharya D Goldrath AW Weissman IL Benoist C Mathis D. Memory T and memory b cells share a transcriptional program of self-renewal with long-term hematopoietic stem cells. Proc Natl Acad Sci U.S.A. (2006) 103(9):3304–9. doi: 10.1073/pnas.0511137103 PMC1413911 16492737

[21] Zheng L Qin S Si W Wang A Xing B Gao R. Pan-cancer single-cell landscape of tumor-infiltrating T cells. Science (2021) 374(6574):abe6474. doi: 10.1126/science.abe6474 34914499

[22] Chiu BC Martin BE Stolberg VR Chensue SW. Cutting edge: Central memory Cd8 T cells in aged mice are virtual memory cells. J Immunol (2013) 191(12):5793–6. doi: 10.4049/jimmunol.1302509 PMC3858473 24227783

[23] Renkema KR Li G Wu A Smithey MJ Nikolich-Zugich J. Two separate defects affecting true naive or virtual memory T cell precursors combine to reduce naive T cell responses with aging. J Immunol (2014) 192(1):151–9. doi: 10.4049/jimmunol.1301453 PMC3925780 24293630

[24] Palmer E Drobek A Stepanek O. Opposing effects of actin signaling and lfa-1 on establishing the affinity threshold for inducing effector T-cell responses in mice. Eur J Immunol (2016) 46(8):1887–901. doi: 10.1002/eji.201545909 27188212

[25] Schober K Voit F Grassmann S Muller TR Eggert J Jarosch S. Reverse tcr repertoire evolution toward dominant low-affinity clones during chronic cmv infection. Nat Immunol (2020) 21(4):434–41. doi: 10.1038/s41590-020-0628-2 32205883

[26] Murata S Sasaki K Kishimoto T Niwa S Hayashi H Takahama Y. Regulation of Cd8+ T cell development by thymus-specific proteasomes. Science (2007) 316(5829):1349–53. doi: 10.1126/science.1141915 17540904

[27] Xing Y Jameson SC Hogquist KA. Thymoproteasome subunit-Beta5t generates peptide-mhc complexes specialized for positive selection. Proc Natl Acad Sci U.S.A. (2013) 110(17):6979–84. doi: 10.1073/pnas.1222244110 PMC3637736 23569244

[28] Nitta T Murata S Sasaki K Fujii H Ripen AM Ishimaru N. Thymoproteasome shapes immunocompetent repertoire of Cd8+ T cells. Immunity (2010) 32(1):29–40. doi: 10.1016/j.immuni.2009.10.009 20045355

[29] Bartleson JM Viehmann Milam AA Donermeyer DL Horvath S Xia Y Egawa T. Strength of tonic T cell receptor signaling instructs T follicular helper cell-fate decisions. Nat Immunol (2020) 21(11):1384–96. doi: 10.1038/s41590-020-0781-7 PMC7578106 32989327

[30] Kwesi-Maliepaard EM Jacobs H van Leeuwen F. Signals for antigen-independent differentiation of memory Cd8(+) T cells. Cell Mol Life Sci (2021) 78(19-20):6395–408. doi: 10.1007/s00018-021-03912-9 PMC8558200 34398252

[31] Schaupp L Muth S Rogell L Kofoed-Branzk M Melchior F Lienenklaus S. Microbiota-induced type I interferons instruct a poised basal state of dendritic cells. Cell (2020) 181(5):1080–+. doi: 10.1016/j.cell.2020.04.022 32380006

[32] Mandl JN Monteiro JP Vrisekoop N Germain RN. T Cell-positive selection uses self-ligand binding strength to optimize repertoire recognition of foreign antigens. Immunity (2013) 38(2):263–74. doi: 10.1016/j.immuni.2012.09.011 PMC3785078 23290521

[33] Schwarzer M Makki K Storelli G Machuca-Gayet I Srutkova D Hermanova P. Lactobacillus plantarum strain maintains growth of infant mice during chronic undernutrition. Science (2016) 351(6275):854–7. doi: 10.1126/science.aad8588 26912894

[34] Shen FW Saga Y Litman G Freeman G Tung JS Cantor H. Cloning of ly-5 cdna. Proc Natl Acad Sci U.S.A. (1985) 82(21):7360–3. doi: 10.1073/pnas.82.21.7360 PMC391344 3864163

[35] Kurts C Miller JFAP Subramaniam RM Carbone FR Heath WR. Major histocompatibility complex class I-restricted cross-presentation is biased towards high dose antigens and those released during cellular destruction. J Exp Med (1998) 188(2):409–14. doi: 10.1084/jem.188.2.409 PMC2212442 9670054

[36] Hogquist KA Jameson SC Heath WR Howard JL Bevan MJ Carbone FR. T-Cell receptor antagonist peptides induce positive selection. Cell (1994) 76(1):17–27. doi: 10.1016/0092-8674(94)90169-4 8287475

[37] Shinkai Y Rathbun G Lam KP Oltz EM Stewart V Mendelsohn M. Rag-2-Deficient mice lack mature lymphocytes owing to inability to initiate V(D)J rearrangement. Cell (1992) 68(5):855–67. doi: 10.1016/0092-8674(92)90029-c 1547487

[38] Fink PJ Swan K Turk G Moore MW Carbone FR. Both intrathymic and peripheral selection modulate the differential expression of V-Beta-5 among Cd4+ and Cd8+ T-cells. J Exp Med (1992) 176(6):1733–8. doi: 10.1084/jem.176.6.1733 PMC2119442 1334117

[39] Kasparek P Krausova M Haneckova R Kriz V Zbodakova O Korinek V. Efficient gene targeting of the Rosa26 locus in mouse zygotes using tale nucleases. FEBS Lett (2014) 588(21):3982–8. doi: 10.1016/j.febslet.2014.09.014 25241166

[40] Lo WL Shah NH Ahsan N Horkova V Stepanek O Salomon AR. Lck promotes Zap70-dependent lat phosphorylation by bridging Zap70 to LAT. Nat Immunol (2018) 19(7):733–41. doi: 10.1038/s41590-018-0131-1 PMC6202249 29915297

[41] Ruedl C Khameneh HJ Karjalainen K. Manipulation of immune system *Via* immortal bone marrow stem cells. Int Immunol (2008) 20(9):1211–8. doi: 10.1093/intimm/dxn079 18644831

[42] Daniels MA Jameson SC. Critical role for Cd8 in T cell receptor binding and activation by Peptide/Major or histocompatibility complex multimers. J Exp Med (2000) 191(2):335–45. doi: 10.1084/jem.191.2.335 PMC2195759 10637277

[43] Howe KL Achuthan P Allen J Allen J Alvarez-Jarreta J Amode MR. Ensembl 2021. Nucleic Acids Res (2021) 49(D1):D884–d91. doi: 10.1093/nar/gkaa942 PMC7778975 33137190

[44] Zheng GX Terry JM Belgrader P Ryvkin P Bent ZW Wilson R. Massively parallel digital transcriptional profiling of single cells. Nat Commun (2017) 8:14049. doi: 10.1038/ncomms14049 28091601 PMC5241818

[45] Lefranc MP Pommie C Kaas Q Duprat E Bosc N Guiraudou D. Imgt unique numbering for immunoglobulin and T cell receptor constant domains and ig superfamily c-like domains. Dev Comp Immunol (2005) 29(3):185–203. doi: 10.1016/j.dci.2004.07.003 15572068

[46] Bolotin DA Poslavsky S Mitrophanov I Shugay M Mamedov IZ Putintseva EV. Mixcr: Software for comprehensive adaptive immunity profiling. Nat Methods (2015) 12(5):380–1. doi: 10.1038/nmeth.3364 25924071

[47] Hao Y Hao S Andersen-Nissen E Mauck WM 3rd Zheng S Butler A. Integrated analysis of multimodal single-cell data. Cell (2021) 184(13):3573–87.e29. doi: 10.1016/j.cell.2021.04.048 34062119 PMC8238499

[48] Ashburner M Ball CA Blake JA Botstein D Butler H Cherry JM. Gene ontology: Tool for the unification of biology. the gene ontology consortium. Nat Genet (2000) 25(1):25–9. doi: 10.1038/75556 PMC3037419 10802651

