# Supplementary material for: Self-reactivity of CD8 T-cell clones determines their differentiation status rather than their responsiveness in infections
Source: Front Immunol. 2022 Oct 6;13:1009198. doi: 10.3389/fimmu.2022.1009198 (PMC9582129; doi:10.3389/fimmu.2022.1009198)
Supplement: Supplementary file 2 [file DataSheet_1.pdf]

Fig. S1

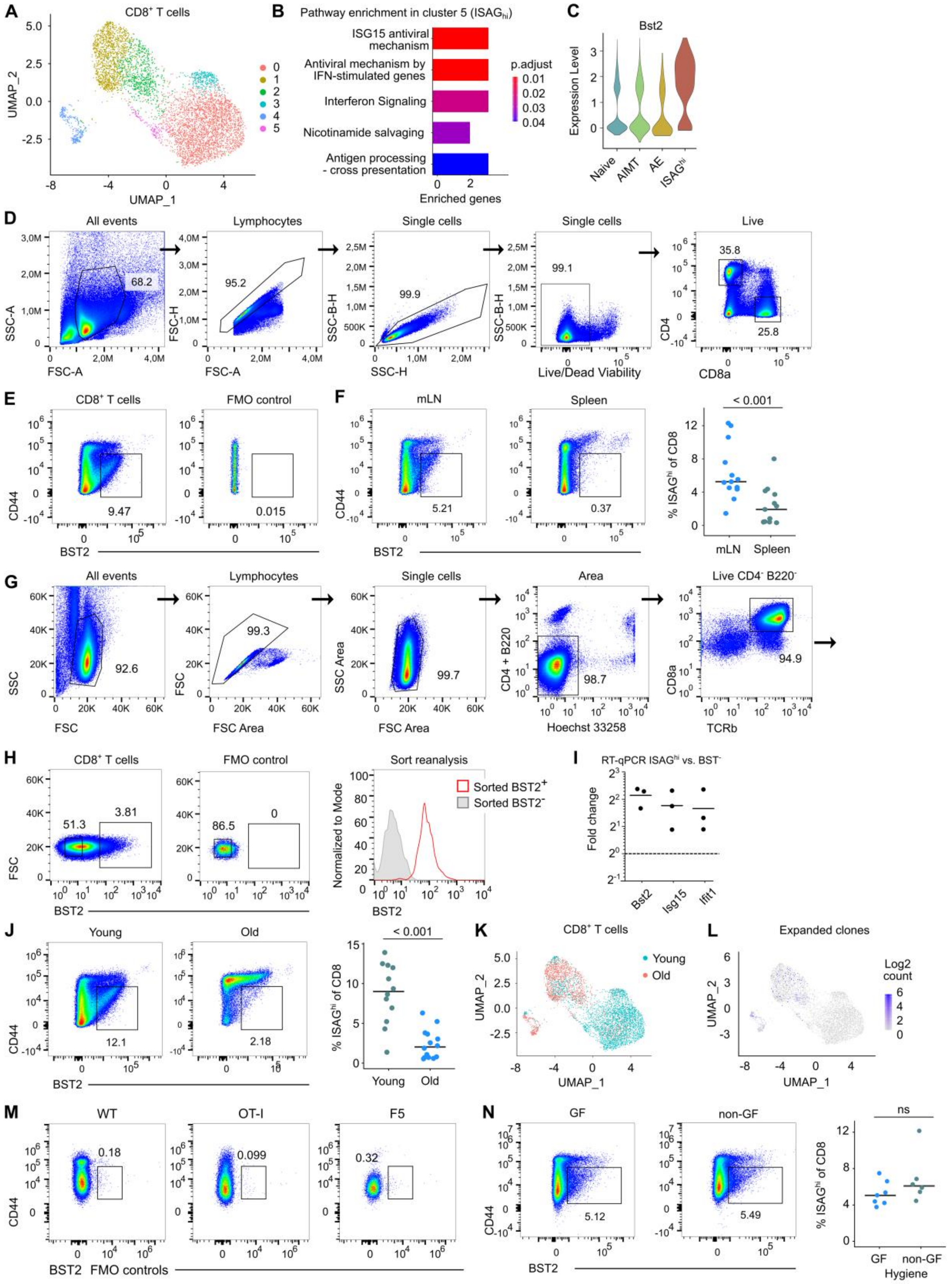
A
CD8⁺ T cells
UMAP_1
UMAP_2
B
Pathway enrichment in cluster 5 (ISAGhi)
ISG15 antiviral mechanism
Antiviral mechanism by IFN-stimulated genes
Interferon Signaling
Nicotinamide salvaging
Antigen processing - cross presentation
Enriched genes
p.adjust
C
Bst2
Expression Level
Naive
AIMT
AE
ISAGhi
D
All events
Lymphocytes
Single cells
Single cells
Live
SSC-A
FSC-A
FSC-H
SSC-B-H
SSC-H
Live/Dead Viability
CD4
CD8a
E
CD8⁺ T cells
FMO control
CD44
BST2
F
mLN
Spleen
% ISAGhi of CD8
< 0.001
G
All events
Lymphocytes
Single cells
Area
Live CD4⁻ B220⁻
SSC
FSC
FSC Area
SSC Area
CD4 + B220
Hoechst 33258
CD8a
TCRb
H
CD8⁺ T cells
FMO control
Sort reanalysis
Sorted BST2⁺
Sorted BST2⁻
Normalized to Mode
FSC
BST2
I
RT-qPCR ISAGhi vs. BST⁻
Fold change
Bst2
Isg15
Ifit1
J
Young
Old
% ISAGhi of CD8
< 0.001
K
CD8⁺ T cells
Young
Old
L
Expanded clones
Log2 count
M
WT
OT-I
F5
BST2 FMO controls
N
GF
non-GF
ns
Hygiene

Supplementary Figure 1. (A) UMAP projection of sorted CD8$^+$ T cells from peripheral blood of C57BL6/J mice processed by scRNAseq. Cells are colored by clusters. **(B)** Reactome pathways significantly enriched in ISAG^{hi} cells (cluster 5). **(C)** The violin plots show the expression of *Bst2* in naïve, AIMT, AE, and ISAG^{hi} cells. **(D-F)** Flow cytometry analysis of CD44^{low} BST2$^+$ ISAG^{hi} T cells in WT mice. **(D)** A representative gating strategy for experiments showed in Fig. 1 and in this figure. **(E)** A representative gating of ISAG^{hi} T cells as CD44^{low} BST2$^+$ and the full minus one staining (FMO) control. The FMO control was stained with secondary anti-biotin antibody without the primary BST2-biotin antibody. **(F)** The percentage of ISAG^{hi} T cells of viable CD8$^+$ T cells in the mLN and the spleen. n = 13 mice in three independent experiments. The statistical analysis was performed using the two-tailed Mann Whitney U test. **(G-H)** A representative gating strategy for sorting BST2$^+$ and BST2$^-$ T cells by FACS. **(G)** Gating of viable CD8$^+$ T cells. **(H)** The sorting gates for BST2$^+$ and BST2$^-$ T cells (left). FMO control stained with secondary anti-biotin antibody without the primary BST2-biotin antibody (middle). Reanalysis of FACS-sorted BST2$^+$ and BST2$^-$ cells (right). A representative experiment out of three in total. **(I)** Relative expression of *Bst2*, *Isg15*, and *Ifit1* in sorted BST2$^+$ or BST2$^-$ T cells measured by qPCR. C_T values of selected genes were normalized to the geometric mean of the C_T values of reference genes *Gapdh*, *Tubb2a*, and *Eef1a1*. The expression levels in BST2$^+$ are normalized to those of BST2$^-$ (=1). The horizontal line represents the mean. Three biological replicates. **(J)** Percentage of ISAG^{hi} T cells of viable CD8$^+$ T cells in the mLN of young (7-12 weeks old) and old (76-81 weeks old) mice. Young: n = 13, old: n = 15 mice in three independent experiments. **(K)** UMAP projection showing the age of mice from which the cells were isolated (young = 8 weeks old, old = 88 weeks old). **(L)** UMAP projection showing the clonal expansion as log2 count of other cells with the same TCRα CDR3 and TCRβ CDR3 nucleotide sequences. Unique clones are grey. **(M)** FMO controls (only secondary anti-biotin antibody without the primary BST2-biotin antibody) for the experiment shown in Fig. 1L. A representative experiment out of three in total. **(N)** Percentage of ISAG^{hi} T cells of viable CD8$^+$ T cells in the mLN of young mice maintained in germ-free (GF) or non-germ-free (SPF or conventional) facilities. GF: n = 7, non-GF: n = 6 mice in two independent experiments.

Fig. S2

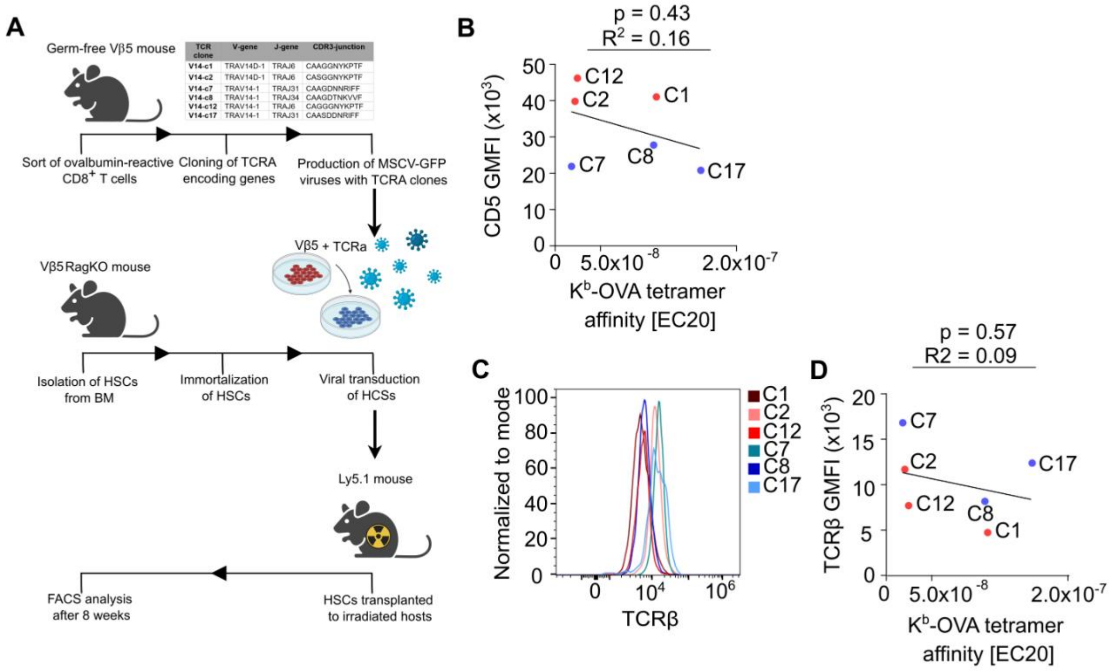

Supplementary Figure 2. (A) Scheme of generation retrogenic clonal populations with indicated CDR3 sequences of particular clones. **(B)** Linear fit of CD5 gMFI vs K^b-OVA tetramer affinity (EC20), determined in the experiment in Figure 2C. **(C)** Expression of surface TCRβ in LN T cells was measured by flow cytometry. A representative experiment out of three in total.**(D)** Linear fit of TCRβ gMFI vs K^b-OVA tetramer affinity (EC20), determined in the experiment in Figure 2C. The Pearson correlation coefficient and p value is shown.

Fig. S3

A

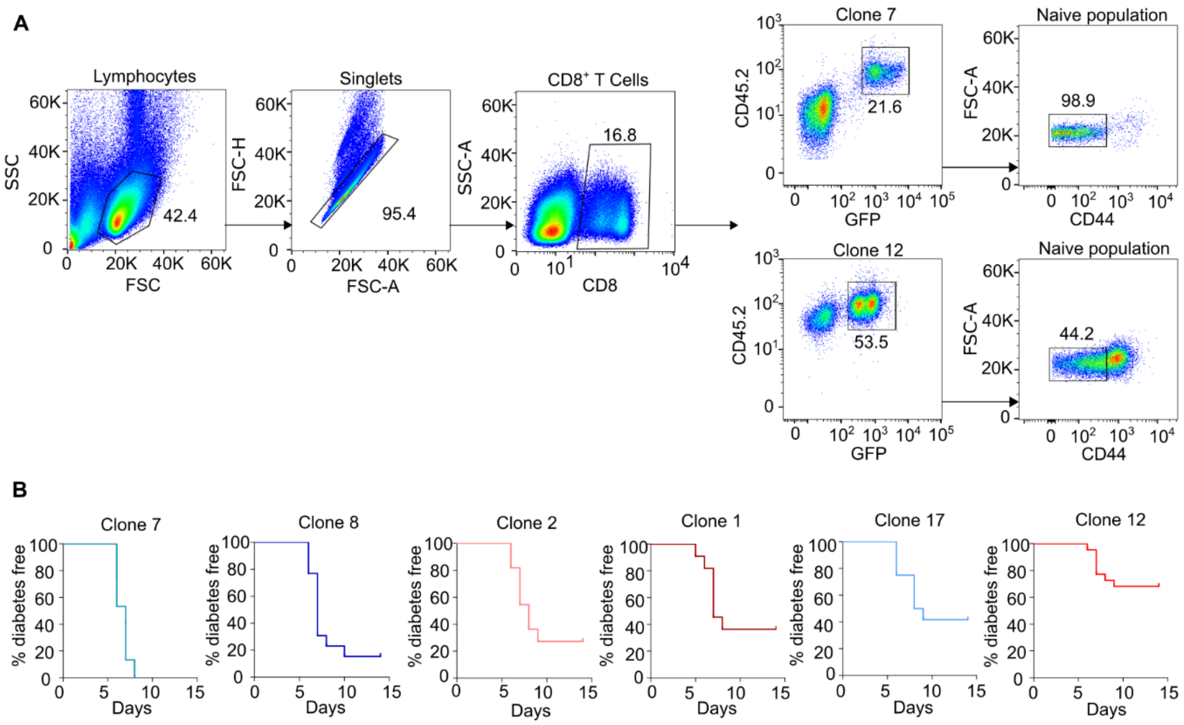

C

TCR clone	V-gene	J-gene	CDR3-junction
TCR8	TRBV31	TRBJ2-7	CASSPRASNYTF
TCR28	TRBV31	TRBJ2-7	CASSPRASDYTF

D

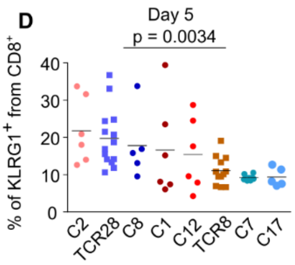

Supplementary Figure 3. (A) Gating strategy for sorting of the naive monoclonal T cells from transplanted Ly5.1 mice. (B) The percentage of diabetes-free mice (i.e., with urine glucose < 1000 mg/dl) in time after the adoptive transfer of the indicated T-cell clone and priming with Lm-OVA on day 0. The same experiment as shown in Fig. 3B. n = 11-22 from three (C1, C2, C8, C17) or four (C7, C12) independent experiments.

(C) CDR3 sequences of TCR8 and TCR28. **(D)** The percentage of $KLRG1^+$ cells among adoptively transferred monoclonal T cells five days after the Lm-OVA infection was determined by flow cytometry (corresponding to the experiments shown in Figure 3G). Mean + SEM. n = 6-12 per group from three independent experiments. The statistical analysis was performed using the Kruskal-Wallis test.

Fig. S4

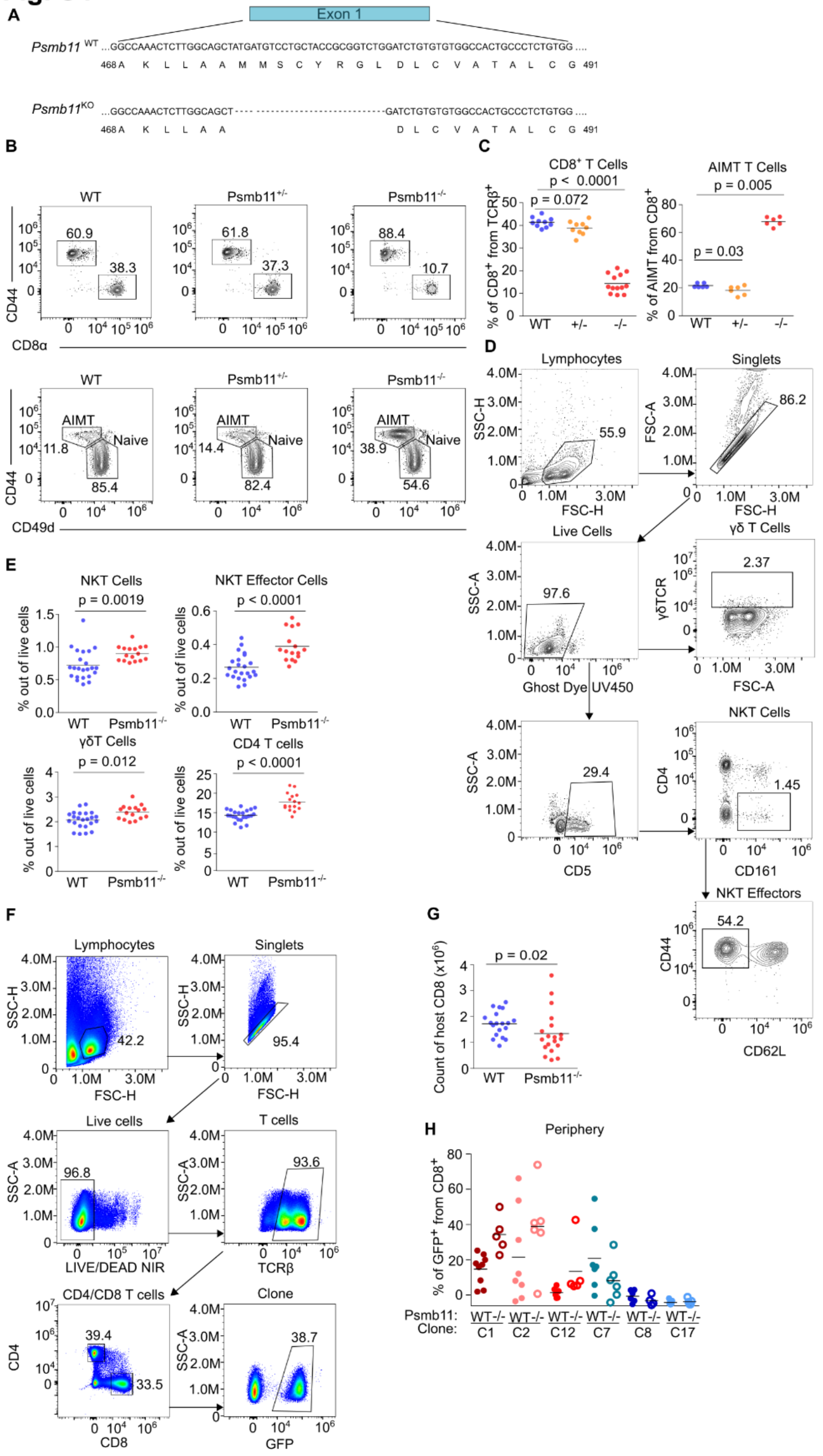
A
Exon 1
Psmb11 WT ...GGCCAAACTCTTGGCAGCTATGATGTCCTGCTACCGCGGTCTGGATCTGTGTGTGGCCACTGCCCTCTGTGG....
468 A K L L A A M M S C Y R G L D L C V A T A L C G 491
Psmb11KO ...GGCCAAACTCTTGGCAGCT----GATCTGTGTGTGGCCACTGCCCTCTGTGG....
468 A K L L A A D L C V A T A L C G 491
B
WT
Psmb11+/-
Psmb11-/-
CD44
CD8α
60.9
38.3
61.8
37.3
88.4
10.7
AIMT
Naive
11.8
85.4
14.4
82.4
38.9
54.6
CD49d
C
CD8+ T Cells
p < 0.0001
p = 0.072
% of CD8+ from TCRβ+
AIMT T Cells
p = 0.005
p = 0.03
% of AIMT from CD8+
WT
+/-
-/-
D
Lymphocytes
SSC-H
FSC-H
55.9
Singlets
FSC-A
86.2
Live Cells
SSC-A
97.6
Ghost Dye UV450
γδ T Cells
γδTCR
2.37
29.4
CD5
NKT Cells
CD4
1.45
CD161
NKT Effectors
CD44
54.2
CD62L
E
NKT Cells
p = 0.0019
NKT Effector Cells
p < 0.0001
γδT Cells
p = 0.012
CD4 T cells
p < 0.0001
% out of live cells
WT
Psmb11-/-
F
Lymphocytes
42.2
Singlets
95.4
Live cells
96.8
LIVE/DEAD NIR
T cells
93.6
TCRβ
CD4/CD8 T cells
39.4
33.5
CD8
Clone
38.7
GFP
G
p = 0.02
Count of host CD8 (x10^6)
WT
Psmb11-/-
H
Periphery
% of GFP+ from CD8+
Psmb11:
Clone:
WT -/-
C1
C2
C12
C7
C8
C17

Supplementary Figure S4. (A) Scheme of targeting of the *Psmb11* locus for the generation of the *Psmb11*$^{-}$ allele by CRISPR/Cas9. **(B)** Representative flow cytometry plots showing the percentage of CD8^{+} and CD4^{+} T cells among TCRβ^{+} splenocytes (top) and the percentage of AIMT (CD44^{+} CD49^{-}) cells among viable CD8^{+} TCRβ^{+} splenocytes (bottom) from WT, *Psmb11*$^{-/-}$ mice, and heterozygous mice. A representative experiment out of three in total. **(C)** The quantification of the frequency of CD8^{+} T cells and AIMT cells from isolated splenocytes in indicated mice. n = 10-13 in three independent experiments. The statistical significance was calculated using the two-tailed Mann Whitney U test. **(D)** A representative gating strategy for the analysis of γδ T cells, NKT cells and NKT effector cells in the spleen. **(E)** The percentage of NKT cells, NKT effector cells, γδ T cells, and CD4^{+} T cells from all live cells in the spleens of WT and *Psmb11*$^{-/-}$ mice. n = 16–22 in three independent experiments. The statistical significance was calculated using the two-tailed Mann Whitney U test. Mean. **(F)** A representative gating strategy for the analysis of bone marrow chimeras in WT and *Psmb11*$^{-/-}$ recipient mice. **(G)** Absolute numbers of LN CD8^{+} T cells from WT and *Psmb11*$^{-/-}$ recipients. n = 19 in three independent experiments. The statistical analysis was performed using the Mann-Whitney U test. **(H)** Percentage of the donor cells (GFP^{+}) from all six clones in the LN of WT and *Psmb11*$^{-/-}$ recipients 8 weeks after the bone marrow transplantations. Median. n = 4-8 mice per group from three independent experiments.

Supplementary Table 1

Differentially expressed genes in clusters 0-5 in the scRNAseq analysis shown in Fig. S1A. The table columns show: the gene name; p-value; average fold change between the cells in the cluster and other cells (log2); frequency of cells with detected expression of the gene in the cluster; frequency of cells with detected expression of the gene outside the cluster; adjusted p value; number of the cluster. The table rows show individual differentially expressed genes.
